# Psychosocial Impact of Facial Paralysis Following Neck Dissection: A Literature Review

**DOI:** 10.7759/cureus.72104

**Published:** 2024-10-22

**Authors:** Arthur P Gatti, Matheus T Ribeiro, Flavio C Hojaij

**Affiliations:** 1 Head and Neck Surgery, Federal University of São Paulo, São Paulo, BRA; 2 Surgery, Hospital das Clínicas da Faculdade de Medicina da Universidade de São Paulo, São Paulo, BRA

**Keywords:** facial nerve paralysis, facial palsy, neck dissection, psychosocial impact, quality of life (qol)

## Abstract

Facial paralysis, a common complication of neck dissection due to facial nerve injuries, results not only in the loss of facial mimicry but also significantly affects patients’ quality of life, particularly in terms of psychosocial perception - an aspect often overlooked by medical teams. This study aims to evaluate the psychosocial impact and perceptions of patients who developed lower third facial paralysis following marginal mandibular nerve injury during neck dissection. A total of 445 postoperative patients who underwent head and neck tumor resection with neck dissection were assessed, of which 217 experienced some degree of facial paralysis. The impact on quality of life varied depending on the assessment scale used, but most patients with lower third facial paralysis reported a reduction in their quality of life and negative effects on their perception of social inclusion. This paralysis, coupled with the stigma of cancer, leads to difficulties in understanding their societal role and concerns about how their social environment perceives them, particularly in social settings.

## Introduction and background

The facial nerve, the seventh pair of cranial nerves, serves an exclusively motor function after passing through the stylomastoid foramen. It has five primary branches: temporal, zygomatic, buccal, marginal of the mandible, and cervical.

The surgical performance of neck dissection for treating metastases in head and neck surgery is a routine therapeutic approach for cancer management [1,2]. Advances in this technique have enhanced patient safety, reducing the risks of functional sequelae while preserving the oncological principles of the procedure [2-4].

Studies on complications arising from neck dissection describe injuries to the marginal nerve of the mandible, a branch of the facial nerve, as one of the most common sequelae of this procedure. Although these injuries are not life-threatening, they can compromise the patient’s quality of life and negatively affect health perception [1-7]. Anatomical variations of this nerve, coupled with the lack of an objective reference point for its location, often complicate its identification during surgery. This difficulty or even the effort to locate the structure can result in temporary or permanent injury, causing paralysis of the facial muscles responsible for mouth corner movement [4,8,9].

Such injuries are reported in approximately 7.3-16% of neck dissection cases, particularly when lymph nodes in levels I and II are involved. However, these figures may be underestimated as data often come from retrospective studies [1,2]. Since surgeons typically focus on oncological outcomes, these consequences tend to be underestimated during follow-up, and their impact on patients’ lives is frequently overlooked [2,5,7].

Despite patients often reporting neuropraxia of the mandibular branch in routine consultations, the psychosocial effects and quality of life implications of this complication remain underexplored in the medical literature [2,3]. Therefore, it is crucial to evaluate whether the patient’s perception of lower third facial paralysis indeed detracts from their quality of life and has a psychosocial impact on their daily routine.

## Review

Materials and methods

**Figure 1:**
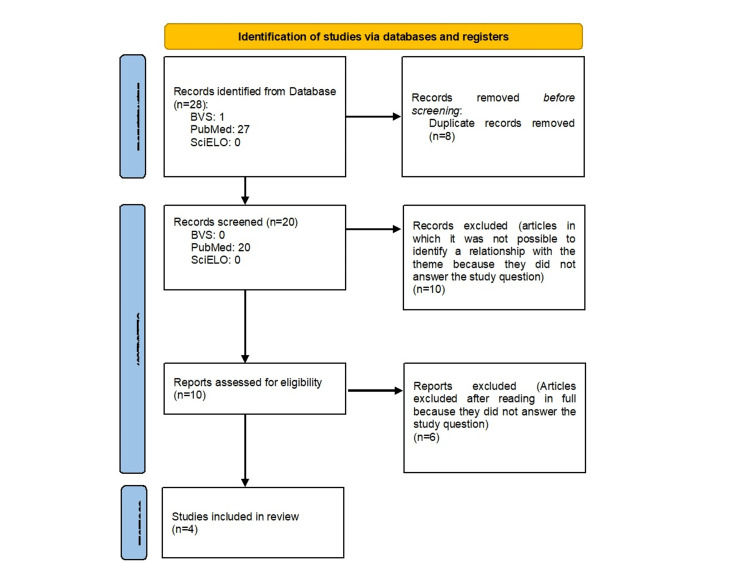
PRISMA flowchart PRISMA, Preferred Reporting Items for Systematic reviews and Meta-Analyses

A literature review was conducted using the Preferred Reporting Items for Systematic reviews and Meta-Analyses (PRISMA) reporting guidelines (Figure 1) through PubMed, BVS, and SciELO, incorporating the following descriptors: “neck dissection,” “facial palsy,” “marginal mandibular nerve injuries,” “life change events,” and “psychology.” Initially, 28 articles from 1981 to 2023 were selected. Articles that did not address nonneoplastic surgical aspects or involved peripheral neurological injuries unrelated to the surgical procedure (e.g., inflammatory, infectious, or rheumatological causes) were excluded. Of the 28 articles, 24 were excluded based on these criteria to avoid bias in analyzing nerve injuries and their associated postoperative psychosocial impact.

Among the four selected articles, a total of 445 patients were analyzed, of whom 217 had undergone head and neck tumor resection. Psychological and social factors were assessed using various scales to evaluate patient perception and the impact of facial paralysis on quality of life.

Results

**Figure 2:**
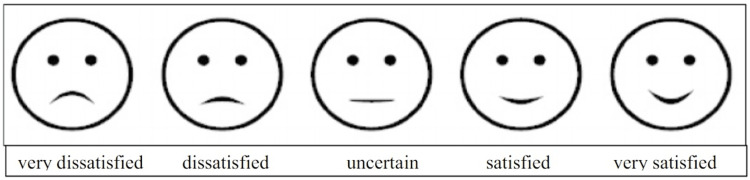
Five-point Likert scale Adapted from Pieralli et al. (2017) [14]

**Figure 3:**
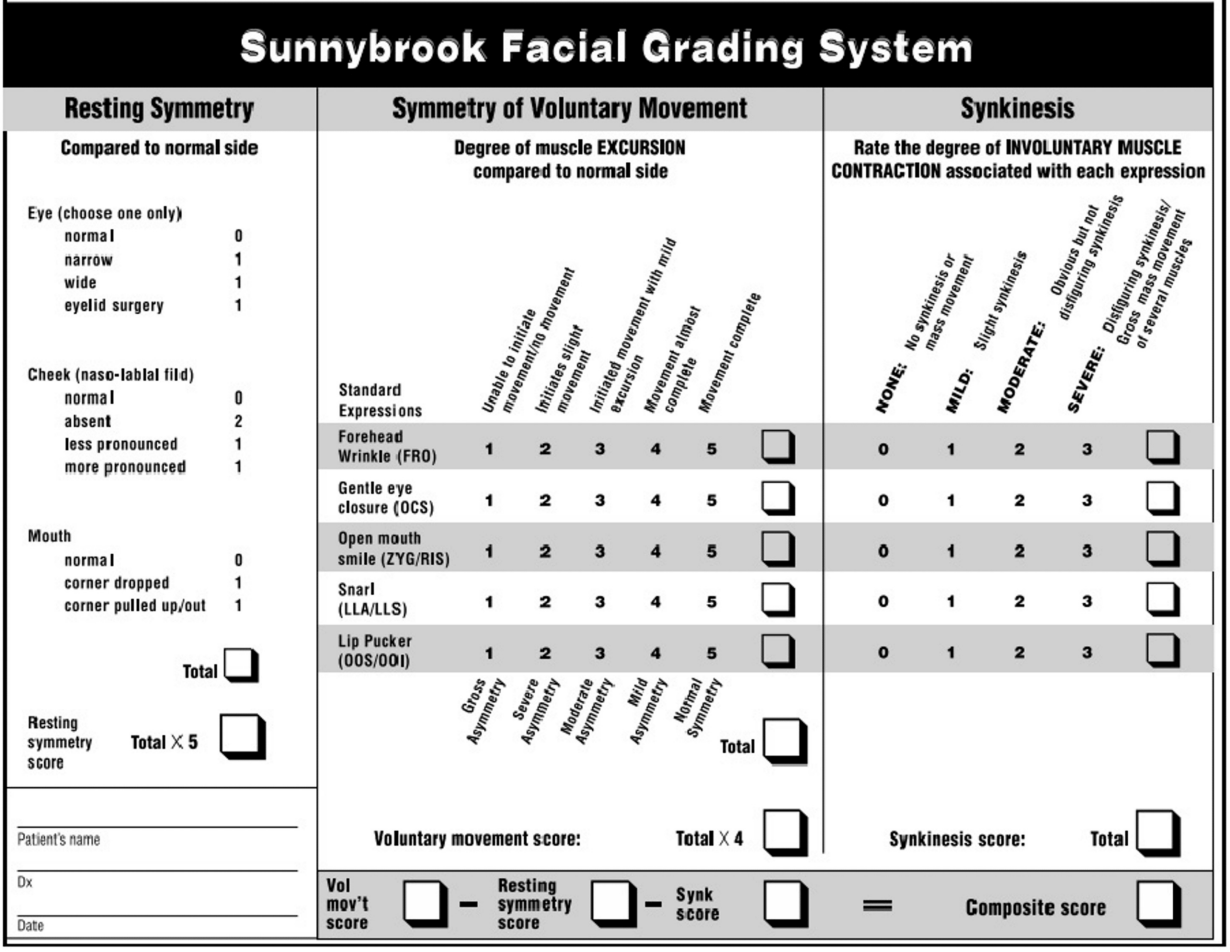
Sydney and Sunnybrook facial grading system Adapted from Mizgajski and Morzy (2019) [16]

**Figure 4:**
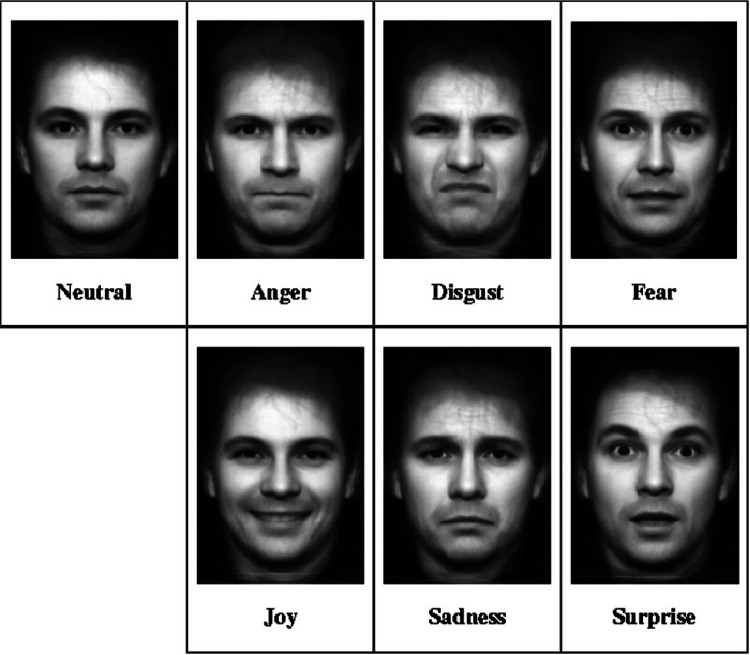
Ekman’s primary emoticons scale Adapted from Falih and Marrogy (2023) [17]

Of the 445 patients analyzed, 217 were selected for having undergone surgeries for tumor resection in the head and neck, experiencing some degree of quality of life loss related to lower third facial paralysis [2,10-12]. All reviewed articles utilized the House-Brackmann scale for the initial classification of facial paralysis severity (Table 1). The studies employed various scales to assess the perceived impact on quality of life, including the Likert Scale (Figure 2), the University of Washington Quality of Life (UWQOL) Scale (Table 2), institutional prompt questions (Table 3), the Sydney and Sunnybrook systems (Figure 3), and Ekman's primary emotions (Figure 4) [2,10-17].

**Table 1:** House-Brackmann facial nerve grading system Adapted from House and Brackmann (1985) [13]

House-Brackmann Facial Nerve Grading System
Grade I - Normal
Normal facial function in all areas
Grade II - Slight Dysfunction
Gross: slight weakness noticeable on close inspection; may have very slight synkinesis
At rest: normal symmetry and tone
Motion: forehead = moderate to good function; eye = complete closure with minimum effort; mouth = slight asymmetry
Grade III - Moderate Dysfunction
Gross: obvious but not disfiguring difference between two sides; noticeable but not severe synkinesis, contracture, and/or hemifacial spasm
At rest: normal symmetry and tone
Motion: forehead = slight to moderate movement; eye = complete closure with effort; mouth = slightly weak with maximum effort
Grade IV - Moderate Severe Dysfunction
Gross: obvious weakness and/or disfiguring asymmetry
At rest: normal symmetry and tone
Motion: forehead = none; eye = incomplete closure; mouth = asymmetric with maximum effort
Grade V - Severe Dysfunction
Gross: only barely perceptive motion
At rest: asymmetry
Motion: forehead = none; eye = incomplete closure; mouth = slight movement
Grade VI - Total Paralysis
No movement

**Table 2:** The Head and Neck Symptom Scale of the UWQOL (UWQOL - RTOG Modification) UWQOL, University of Washington Quality of Life Adapted from Pugh et al. (2017) [15]

The Head and Neck Symptom Scale of the UWQOL Questionnaire (UWQOL - RTOG Modification)		
Each of the following items lists different numbered statements. Think about what each statement says, then place a circle around the one statement that most closely describes how you have been feeling during the past week, including today. Please encircle only one statement for each item.		
1. PAIN		B. Swallowing
A. General		10 I swallow normally.
10 I have no pain.		20 I cannot swallow certain solid foods.
20 There is mild pain; no need for medication.		30 I can only swallow soft foods.
30 I have moderate pain -it requires regular medication (codeine or non-narcotic).		40 I can only swallow liquid foods.
40 I have severe pain controlled only by narcotics.		50 I cannot swallow.
50 I have severe pain, not controlled by medication.		7. SALIVA
B. Mouth		A. Amount
10 I have no pain in my mouth.		10 I have a normal amount of saliva.
20 I have mild pain, but it is not affecting my eating.		20 I have a mild loss of saliva.
30 I have moderate pain that is affecting my eating.		30 I have a moderate loss of saliva.
40 I have severe pain and need medication in order to eat.		40 I have a severe loss of saliva.
50 I have severe pain and cannot eat with the medication.		50 I have no saliva.
C. Throat		B. Consistency
10 I have no pain in my mouth.		10 My saliva has a normal consistency.
20 I have mild pain, but it is not affecting my eating.		20 My saliva is slightly thicker.
30 I have moderate pain that is affecting my eating.		30 My saliva is moderately thicker.
40 I have severe pain and need medication in order to eat.		40 My saliva is extremely thicker.
50 I have severe pain and cannot eat with the medication.		50 I have saliva that dries in my mouth and/or on my lips.
2. DISFIGUREMENT		8. TASTE
10 There is no change in my appearance.		10 I have tasted food normally.
20 The change in my appearance is minor.		20 I can taste most food normally.
30 My appearance bothers me, but I remain active.		30 I can taste some foods normally.
40 I feel significantly disfigured and limit my activities due to my appearance.		40 I can taste few foods normally.
50 I cannot be with people due to my appearance.		50 I cannot taste any foods normally.
3. ACTIVITY		9. SPEECH
10 I am as active as I have ever been.		10 My speech is the same as always.
20 There are times when I can’t keep up my old pace, but not often.		20 I have difficulty saying some words, but I can be understood over the phone.
30 I am often tired and have slowed down my activities, although I still get out.		30 I have moderate difficulty saying some words, and I cannot use the phone.
40 I don’t go out because I don’t have the strength.		40 Only my family and friends can understand me.
50 I am usually in bed or chair and don’t leave home.		50 I cannot be understood.
4. RECREATION/ENTRETEINMENT		10. MUCUS OR PHLEGM
10 There are no limitations to recreation at home or away from home.		A. Amount
20 There are a few things I can’t do but still get out and enjoy life.		10 I have a normal amount of mucus.
30 There are many times I wish I could get out more, but I’m not up to it.		20 I have a mild loss of mucus.
40 There are severe limitations to what I can do; mostly, I stay at home and watch TV.		30 I have a moderate loss of mucus.
50 I can’t do anything enjoyable.		40 I have a severe loss of mucus.
5. EMPLOYMENT		50 I have no mucus.
10 I work full time.		B. Consistency
20 I have a part-time but permanent job.		10 My mucus has a normal consistency.
30 I only have occasional employment.		20 My mucus is slightly thicker.
40 I am unemployed.		30 My mucus is moderately thicker.
50 I am retired (circle one below):		40 My mucus is extremely thicker.
51 Not related to cancer treatment		50 I have no mucus.
52 Due to cancer treatment		Please describe any other issues (medical or nonmedical) that are important to your quality of life and have not been adequately addressed by our questions:
6. EATING		
A. Chewing		__________________________________________________________
10 I can chew as well as ever.		__________________________________________________________
20 I have slight difficulty chewing solid foods.		__________________________________________________________
30 I have moderate difficulty chewing solid foods.		__________________________________________________________
40 I can only chew soft foods.		
50 I cannot chew soft foods.		

**Table 3:** Institutional prompt questions of patient values and perceptions of facial nerve palsy Adapted from Hasmat et al. (2023) [11]

Institutional prompt questions
1. What has been your experience of facial paralysis?
2. What aspect(s) of the paralysis has been most difficult to deal with, and why?
3. How have you managed to help the situation?
4. Are you satisfied with the help you have received so far?

In the first article analyzed, among the 66 related patients (with 85 neck dissections performed), 18% initially exhibited some degree of facial paralysis according to the House-Brackmann scale. When assessed using the Likert Scale (Figure 2), 50% of the patients did not report any changes in facial motility, particularly regarding their smiles. Additionally, 41% experienced minimal limitations in motility, 5% reported a significant change that did not affect their quality of life, and 3% indicated that the impact was so severe that their disfigurement greatly affected their quality of life. Notably, no patient reported feeling unable to socialize after surgery [2].

In another article, following tumor resection surgery in 278 patients, 114 individuals (41%) reported some degree of psychological limitation due to facial paralysis, as measured by the UWQOL scale (score <75). Among these patients, 220 (79.13%) had undergone neck dissection related to their complaints about facial motility. Specifically, 50% had surgical manipulation at levels 3-4 (76 out of 153 patients), 41% (nine out of 22) had neck dissection at level 5, 38% (15 out of 39) underwent bilateral surgery, and 50% (three out of six) were treated with radical or modified radical neck dissection. The impact on the lower levels of the scale was not analyzed; instead, only the absolute number of patients experiencing a negative impact on their quality of life was reported [10].

In another study examining patient perceptions and values, a standardized institutional questionnaire (Table 2) was employed, which included five areas of analysis: (1) ocular symptoms; (2) fear of judgment and social isolation; (3) aversion to potential new invasive surgeries; (4) need for simplified multidisciplinary care; and (5) lack of public awareness. Among the 13 patients analyzed, seven reported complaints related to the oral site, while 12 experienced ocular complaints, with six indicating that ocular issues significantly impacted their quality of life. Additionally, 11 patients expressed concerns that their paralysis would adversely affect their social lives and public perception, particularly in the workplace. Seven patients were unwilling to undergo invasive facial surgeries again; however, all would accept minimally invasive options. Three patients indicated a need for multidisciplinary interventions, primarily group support therapies. Furthermore, four patients were unaware of the psychological impact that their treatment could have on their lives [11].

Another cross-sectional study analyzed 24 patients with facial paralysis following oncological surgical procedures, using both Ekman’s primary emotions scale and the Likert scale. The study focused on the impact of facial motility on emotional expression and quality of life. Patients were asked whether their facial expressions accurately conveyed the emotions they intended to express - namely joy, displeasure, surprise, anger, sadness, and fear - according to Ekman’s primary emotions scale. Among the participants, 11 patients (45%) reported difficulty expressing their feelings, with surprise being the most affected emotion (63.63%) [12,18]. When evaluating the Sydney and Sunnybrook systems, a significant difference was observed between the effective and non-effective patient groups concerning the expression of joy, surprise, and sadness. Patients reported challenges due to decreased movement (74%), synkinesis (48%), dissatisfaction with their facial appearance (13%), and asymmetry (3%) [12].

Discussion

The patient’s perception of the impact on their quality of life is a topic that is underaddressed in the literature, with the number of publications significantly lacking considering the importance of the subject. Most studies concerning facial paralysis following surgical treatments in the head and neck - especially neck dissection - primarily focus on oncological outcomes, tumor recurrences, and adjuvant therapies [2,10]. The incidence of facial paralysis in the studied population is clearly underestimated, as it is often analyzed without standardized classification criteria, and the medical team may interpret aesthetic results differently than patients, who are more focused on therapeutic outcomes [11,12].

The prior understanding of both the patient and their family greatly influences how these individuals cope with the facial motor sequelae [5,19,20]. Some authors have noted that patients who developed facial paralysis after plastic procedures experienced better psychological outcomes when they received clearer explanations about their facial sequelae [20].

This study has a bias related to the varying scales used to analyze the impact of facial paralysis on patients’ quality of life. The absence of a standardized framework complicates efforts for research centers to unify their evaluations, rendering real comparative analysis impossible. Nonetheless, it is feasible to extrapolate results and draw parallels to characterize this impact on the studied group, particularly at the extremes - those without changes in quality of life and those whose experiences hinder their social engagement [21].

The limitations in self-expression lead to a significant loss of quality of life, often isolating patients in their work and family environments, which can contribute to increased rates of depression and a higher risk of suicide [2,20,21]. This impact should be considered not only after the sequelae arise but also during the preoperative preparation of the patient, ideally with a multidisciplinary team to provide guidance.

Scales utilized in this study for grading paralysis, such as the House-Brackmann scale, along with scales for both subjective and objective evaluations of patients' perceptions of their facial motor complaints (including the Likert Scale, UWQOL scale, Ekman’s primary emotions, and the Sydney and Sunnybrook systems), have been validated for these purposes. These tools enable the care team to identify and address psychosocial disorders that may develop alongside the oncological treatment.

We found that the perception of lower third facial paralysis negatively impacts the quality of life of these patients, leading to loss of self-esteem and increasing feelings of social segregation. This situation disrupts fundamental family ties during their oncological treatment, diminishes their self-recognition as functional and social individuals, and reduces their acceptance of other therapeutic proposals.

Research examining the true psychological and social impact on individuals who endure not only the underlying disease but also the direct effects of surgical and adjuvant treatments is scarce in medical literature. Therefore, it is crucial to conduct more qualitative and quantitative analyses on this topic to enhance the level of care provided and optimize recovery for these patients, resulting in better quality-of-life outcomes.

## Conclusions

Lower third facial paralysis secondary to injuries of the marginal nerve of the mandible during neck dissection surgery in oncological patients significantly impacts their quality of life, both during and after treatment. Approximately 50% of the analyzed patients who underwent this type of surgery reported some degree of motor complaint and psychosocial impact. Notably, 45% experienced difficulties in expressing feelings through facial mimicry, with 5% of these cases being severe. Although such injuries are common sequelae, they are often underestimated by healthcare professionals. The disfigurement perceived by these patients creates a negative impact on their self-esteem, affecting not only themselves but also their social circles.

The assessment of this impact can vary based on the patient’s prior understanding and the extent of their paralysis. Various scales can be employed to measure this impact; however, the lack of standardization and the low recognition of this negative impact by care teams hinder the effective management of this sequela, exacerbating its effects on patients’ lives.

## References

[1] Dedivitis RA, Guimarães AV, Pfuetzenreiter EG Jr, Castro MA (2011). Neck dissection complications [Article in Portuguese]. Braz J Otorhinolaryngol.

[2] Batstone MD, Scott B, Lowe D, Rogers SN (2009). Marginal mandibular nerve injury during neck dissection and its impact on patient perception of appearance. Head Neck.

[3] Murthy SP, Paderno A, Balasubramanian D (2019). Management of the marginal mandibular nerve during and after neck dissection. Curr Opin Otolaryngol Head Neck Surg.

[4] Nason RW, Binahmed A, Torchia MG, Thliversis J (2007). Clinical observations of the anatomy and function of the marginal mandibular nerve. Int J Oral Maxillofac Surg.

[5] Larsen MH, Lorenzen MM, Boakholdt V (2020). The prevalence of nerve injuries following neck dissections - a systematic review and meta-analysis. Dan Med J.

[6] Petsinis V, Papadogeorgakis N, Evangelou I, Goutzanis L, Pandelidaki E, Alexandridis C (2009). Metastases to supramandibular facial lymph nodes in patients with squamous cell carcinoma of the oral cavity. J Oral Maxillofac Surg.

[7] Nocon CC, Cohen MA, Langerman AJ (2016). Quality of neck dissection operative reports. Am J Otolaryngol.

[8] Mohan R, Brown EN, Borsuk DE, Christy MR, Bojovic B, Rodriguez ED, Dorafshar AH (2014). Revisiting the anatomic relationship of the marginal mandibular nerve and the posterior facial vein: a cadaveric study. Ann Plast Surg.

[9] Yang HM, Kim HJ, Park HW, Sohn HJ, Ok HT, Moon JH, Woo SH (2016). Revisiting the topographic anatomy of the marginal mandibular branch of facial nerve relating to the surgical approach. Aesthet Surg J.

[10] Millsopp L, Brandom L, Humphris G, Lowe D, Stat C, Rogers S (2006). Facial appearance after operations for oral and oropharyngeal cancer: a comparison of casenotes and patient-completed questionnaire. Br J Oral Maxillofac Surg.

[11] Hasmat S, Low TH, Dusseldorp JR (2023). Exploring patient values and perceptions with facial nerve palsy to help guide management: an Australian perspective. Australas J Plast Surg.

[12] Coulson SE, O'dwyer NJ, Adams RD, Croxson GR (2004). Expression of emotion and quality of life after facial nerve paralysis. Otol Neurotol.

[13] House JW, Brackmann DE (1985). Facial nerve grading system. Otolaryngol Head Neck Surg.

[14] Pieralli A, Bianchi C, Longinotti M (2017). Erratum to: Long-term reliability of fractioned CO2 laser as a treatment for vulvovaginal atrophy (VVA) symptoms. Arch Gynecol Obstet.

[15] Pugh SL, Wyatt G, Wong RK (2017). Exploratory factor analysis of NRG Oncology's University of Washington Quality of Life Questionnaire-RTOG modification. J Pain Symptom Manage.

[16] Mizgajski J, Morzy M (2019). Affective recommender systems in online news industry: how emotions influence reading choices. User Model User-Adap Inter.

[17] Falih MO, Marrogy HA (2023). Marginal mandibular nerve weakness due to a giant osteoma: a case report. J Med Case Rep Case Series.

[18] Zourntou SE, Makridis KG, Tsougos CI, Skoulakis C, Vlychou M, Vassiou A (2021). Facial nerve: a review of the anatomical, surgical landmarks and its iatrogenic injuries. Injury.

[19] Vickery LE, Latchford G, Hewison J, Bellew M, Feber T (2003). The impact of head and neck cancer and facial disfigurement on the quality of life of patients and their partners. Head Neck.

[20] Nellis JC, Ishii M, Byrne PJ, Boahene KD, Dey JK, Ishii LE (2017). Association among facial paralysis, depression, and quality of life in facial plastic surgery patients. JAMA Facial Plast Surg.

[21] Fu L, Bundy C, Sadiq SA (2011). Psychological distress in people with disfigurement from facial palsy. Eye (Lond).

